# Molecular Subtyping and Precision Medicine for Pancreatic Cancer

**DOI:** 10.3390/jcm10010149

**Published:** 2021-01-04

**Authors:** Fieke E. M. Froeling, Raffaella Casolino, Antonio Pea, Andrew V. Biankin, David K. Chang, on behalf of Precision-Panc

**Affiliations:** 1 Wolfson Wohl Cancer Research Centre, Institute of Cancer Sciences, University of Glasgow, Garscube Estate, Switchback Road, Bearsden, Glasgow G61 1BD, UK; fiekefroeling@gmail.com (F.E.M.F.); raffaella.casolino@glasgow.ac.uk (R.C.); antonio.pea@univr.it (A.P.); Andrew.Biankin@glasgow.ac.uk (A.V.B.); 2 Edinburgh Cancer Centre, Western General Hospital, NHS Lothian, Crewe Road South, Edinburgh EH4 2XU, UK; 3 Department of Medicine, University and Hospital Trust of Verona of Verona, Piazzale L.A. Scuro 10, 37134 Verona, Italy; 4 Department of Surgery, University and Hospital Trust of Verona, Piazzale L.A. Scuro 10, 37134 Verona, Italy; 5 West of Scotland Pancreatic Unit, Glasgow Royal Infirmary, Glasgow G31 2ER, UK

**Keywords:** pancreatic cancer, pancreatic ductal adenocarcinoma, molecular subtypes, precision medicine, Precision-Panc

## Abstract

Substantial progress in recent years has dramatically increased our knowledge of the molecular basis of cancer, revealing new potential therapeutic targets and paving the way for effective personalised medicine for the treatment of many tumour types. However, pancreatic cancer has been lagging behind in this success and continues to be one of the most lethal solid malignancies. Its molecular heterogeneity and the unselected design of the majority of clinical trials to date can in part explain the reason for our failure to make a significant change in the survival outcomes for patients with pancreatic cancer. A changing paradigm in drug development is required to validate the new molecular taxonomy and to rapidly translate preclinical discovery into clinical trials. Here, we review the molecular subtyping of pancreatic cancer, the challenges in identifying effective treatment regimens according to defined low-prevalence molecular subgroups and we illustrate a new model of translational therapeutic development that was established in the U.K. (Precision-Panc) as a potentially effective solution to improve outcomes for patients with pancreatic cancer.

## 1. Introduction

Advances in next-generation sequencing (NGS) technologies are transforming the way we are diagnosing and treating cancer. Large-scale initiatives from cooperative groups, such as The Cancer Genome Atlas (TCGA) and the International Cancer Genome Consortium (ICGC), have characterised more than 26,000 cancer genomes from a wide range of tumour types, resulting in the identification of multiple genomic cancer drivers that can be therapeutically targeted [1,2,3,4,5,6]. Pioneering examples of molecularly driven treatment are the development of tamoxifen in the seventies to target the oestrogen receptor in breast cancer and the kinase inhibitor imatinib for the treatment of chronic myelogenous leukaemia (CML) carrying the BCL-ABL1 chromosomal translocation [7,8]. Since these early successes, the clinical trial portfolio of matching treatments to genomic alterations is expanding globally and precision medicine approaches are changing the lives of many patients. However, the increased knowledge about the molecular pathology of cancer has highlighted its complexity and poses marked challenges in translating genomic and scientific discoveries into the clinic. This is particularly true for pancreatic cancer (PC), which is one of the most lethal cancers that is predicted to soon become the second leading cause of cancer death [9,10]. Despite increasing knowledge about its molecular landscape, the majority of patients are treated with combination chemotherapy, which has a modest impact on patients’ outcomes at the cost of marked toxicity. Recently, adjuvant therapy with modified FOLFIRINOX (FFX; 5-fluorouracil, leucovorin, oxaliplatin and irinotecan) showed a median disease-free survival of 21.6 months compared to 12.8 months with gemcitabine [11] and has become the standard of care for fit patients with resectable disease. However, this group represents less than 20% of patients, of whom many are not able to tolerate triple-agent chemotherapy postoperatively. For the majority of patients presenting with advanced disease, the best approved regimens are FFX and gemcitabine/nab-paclitaxel, but unfortunately, median survival rates remain less than one year [12,13]. To date, there are no predictive molecular markers that can identify which patients will benefit from FFX versus gemcitabine/nab-paclitaxel, and clinician judgement and the patient’s performance status often dictate the treatment choice. Thus, there is an urgent need to improve outcomes for pancreatic cancer patients, change the current treatment paradigms and work towards a biomarker-driven personalised approach. Here, we review the accumulating knowledge about the molecular pathology of pancreatic cancer and the clinical implications and describe the novel clinical trial platforms that aim to translate clinically relevant genome discoveries into the real world.

## 2. Mutational Landscape of Pancreatic Cancer

Advances in nucleic acid sequencing and large international efforts to analyse the genome, epigenome and transcriptome of pancreatic cancer have resulted in significant improvements in our understanding of its molecular pathology [1,2,3,14,15,16,17,18]. Apart from well-known somatic mutations in *KRAS*, *TP53*, *SMAD4* and *CDKN2A*, each of which is altered in >50% of patients, PC is highly heterogeneous with a myriad of driver and passenger alterations in numerous genes. With the increase in genomic data over time, other genes that mutate at a frequency of 5–10% have been identified, including many epigenetic regulators, such as *KDM6A*, *MLL3* and *ARID1A,* but most mutations occur at a prevalence of <5%, with a median of <1% [15,19]. Recent improvements in molecular biology have also allowed for the understanding of the complex interactions of intra-tumoural signalling that is generated by the (in)activation of the most important oncogenic drivers or tumour suppressors. Specifically, genetic alterations in PC converge in intricate core pathways, which contribute to the hallmarks of cancer, including RAS signalling, the TGF-β pathway, cell cycle control, WNT/Notch signalling, epigenetic regulation and DNA damage repair. Of these, the RAS–MAPK constitutes the most frequently affected pathway: up to 90–95% of PC present with activating *KRAS* mutations and alternative RAS-MAPK pathway alterations are seen in ≈60% of *KRAS* wild-type tumours [18,20]. Although this grouping in signalling pathways is useful in the understanding of tumour biology, the network is highly complex with multilevel interactions and cross-talk between the different molecular cascades. Moreover, recurrent non-coding mutations have also been associated with genes regulating the oncogenic signalling pathways in PC [21]. Thus, these aspects highlight the challenges in the therapeutic inhibition of single gene activations in PC, and may in part explain the failure of many clinical trials investigating this approach.

## 3. Molecular Targets Based on Genomics

### 3.1. Single Genetic Alterations

**Figure 1:**
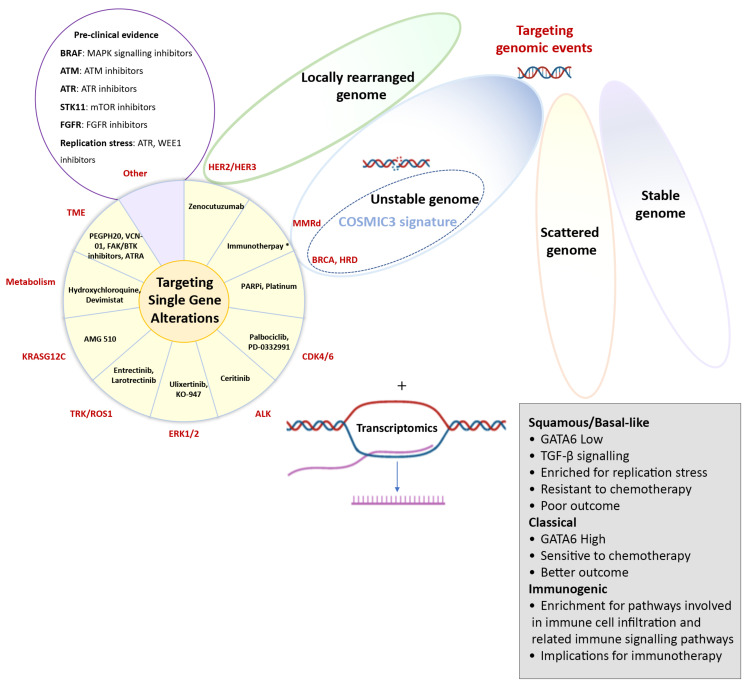
Molecular targets in pancreatic cancer. Potential molecular targets in pancreatic cancer, including therapeutic targeting of single-gene alterations, or possibly larger subgroups that are identified by whole genome sequencing (locally rearranged or unstable genomes), mutational signatures or transcriptomic subtypes. The pie diagram shows specific compounds currently under clinical investigation based on single-gene alterations or targeting the stroma or altered metabolism. However, this list continues to expand and “Other” includes potential other targets based on encouraging preclinical evidence with various drugs with similar mechanisms of action being evaluated.

Many studies have researched both the prognostic and predictive value of single genetic alterations. However, the majority of those have not been independently validated or failed to be successfully translated into routine clinical practice. An important exception is the recent approval of the poly(ADP-ribose) polymerase (PARP) inhibitor olaparib as a first-line maintenance treatment for patients with germline *BRCA*-mutated metastatic PC, representing approximately 2–4% of unselected patients, based on the POLO trial [22]. In this practice-changing study, olaparib treatment after the initial response to first-line platinum chemotherapy resulted in a 3.6-month improvement in median progression free survival (PFS) compared to a placebo (7.4 months vs. 3.8 months; Hazard Ratio (HR) = 0.53 (0.35–0.82), *p* = 0.004) [22]. Other approved therapies for patients with PC include the immune checkpoint inhibitor pembrolizumab, which blocks the interaction between programmed cell death-1 (PD-1) receptor and its ligands PD-L1 and PD-L2 if the tumour demonstrates microsatellite instability (MSI) [23,24] or the tropomyosin receptor kinase (TRK) inhibitors larotrectinib or entrectinib are in the presence of neurotrophic tyrosine/tropomyosin receptor kinase (NTRK) gene fusion [25,26]. Both MSI and NRTK gene fusions can be reliably detected using routine immunohistochemistry, fluorescence in situ hybridisation (FISH) assays or targeted sequencing; however, due to their low prevalence in PC (1–3%), only a very small group of patients is receiving any benefit from these treatment options. Similarly, the FDA recently granted orphan drug designation to zenocutuzumab, a bispecific HER2 and HER3 receptor antibody that blocks binding of the HER3 ligand neuregulin 1 (NRG1) or NRG1-fusion proteins, for patients with previously treated PC harbouring NRG1 gene fusions, which occurs in 0.5–1.5% of cases [27]. Despite the low frequency of most individual genetic alterations, recent studies have shown that 25–40% of PC patients harbour at least one genomic alteration that could potentially be therapeutically targeted [2,28], and a wide range of compounds is currently under investigation (Figure 1). However, to date, the number of pancreatic cancer patients receiving molecularly matched treatment remains <5%, highlighting the challenges of implementing precision medicine in pancreatic cancer [29]. Thus, there is an urgent need to identify larger groups of patients, based on shared molecular events beyond point mutations in coding genes, who are likely to benefit from certain treatments [30].

### 3.2. Genomic Subgroups

In addition to grouping based on altered intracellular signalling pathways, histopathologically similar cancers can be grouped based on other genomic aberrations (Figure 1). A large collaborative effort led by the Sanger institute analysed 4,942,984 mutations from 7042 cancers across 30 different tumour types. By categorising the genome-wide trinucleotide context of single nucleotide variants (SNVs), somatic mutations could be grouped into different mutational signatures representing the mutagenic mechanisms that occur before and during tumour development [31,32]. Several of these COSMIC (Catalogue of Somatic Mutations in Cancer) signatures are associated with ageing, known mutagenic environmental exposures, such as smoking and ultraviolet light, or due to defects in DNA repair. However, for most of the signatures identified, the inciting stimulus has yet to be identified. Mutational signatures known to be important in the biology of PC include older age, BRCA-mediated defects in DNA damage repair, DNA mismatch repair deficiency and a signature associated with the APOBEC family of cytidine deaminases [2,3,31,33]. Individually, these mutational signatures do not yet have clinical utility; however, incorporating them into a comprehensive assay that includes multiple genomic events based on an understanding of the biology is showing promise, in particular, for identifying patients who are likely to benefit from drugs targeting DNA damage repair [3,34].

In addition to patterns of mutational signatures, whole-genome sequencing (WGS) has shown that PC can be classified into four groups based on the frequency and distribution of structural variations of the genome: stable genomes (<50 structural variants per genome), scattered genomes (50–200 structural variants per genome), locally rearranged genomes (>200 structural variants clustered on <3 chromosomes), or unstable genomes (>200 structural variants distributed across the genome) [3]. The potential clinical relevance of this classification involves patients with a locally rearranged or unstable genome. The locally rearranged subtype is characterised by focal amplifications of genes that can be therapeutically targeted, such as *ERBB2* (also known as *HER2*), *FGFR*, *PIK3CA* and *PIK3R3*. Despite being altered in only ≈2% of cases, *ERBB2* drives important biological signalling in PC, is associated with lung and brain dissemination (as opposed to liver metastasis as the most common metastatic site) and can be targeted using widely used *ERBB2* inhibitors [35,36,37]. However, the low prevalence of these alterations in each individual patient continues to limit testing and routine clinical use in PC. The unstable subtype, which is characterised by defects in DNA damage response (DDR) and potentially increased sensitivity to DNA damaging agents, such as platinum-based chemotherapy or PARP inhibition, may yield therapeutic options for a larger subgroup of patients [3,38]. Unstable genomes showed a significant overlap with other surrogate markers of DNA maintenance, in particular, the COSMIC *BRCA* mutational signature and mutations in DDR genes [3]. Furthermore, DNA repair defects extended beyond germline variations in *BRCA*1/2 genes and included other genes that contribute to the DDR signature, such as germline and somatic mutations in *PALB2* and somatic mutations in *BRCA1*, *BRCA2*, *ATM*, *RPA1*, *REV3L*, *XRCC4* and *XRCC*. According to these findings, approximately 24% of PC patients express putative biomarkers of DDR deficiency, and thus, show potential sensitivity to DNA damaging agents; this is a significantly larger group of patients than the currently approved use of olaparib for patients with germline pathogenic variants in BRCA1/2 [3,22]. The use of PARP inhibitors beyond germline *BRCA*-mutated cancer has already shown to be of clinical benefit in other tumour types and multiple clinical trials are currently evaluating this approach in PC [39,40]. In addition, novel DDR inhibitors are being developed, including new potent and relatively selective PARP inhibitors, agents targeting DDR-signalling proteins (*DNA-PK*, *ATM*, *ATR*) and agents causing transient cell cycle delays, such as *WEE-1* and cell-cycle checkpoint inhibitors [41]. These DDR agents are currently under investigation for patients with molecularly selected advanced tumours (as monotherapy and in combinations with other targeted agents or immune checkpoint inhibitors) and represent a potential therapeutic opportunity for PC patients with a DDR signature [42].

## 4. Transcriptomic Subtypes of Pancreatic Cancer

A more recent approach to PC subtyping has been made by analysing the transcriptional networks and several classifications have been produced to date (reviewed in [43]). In 2011, Collisson et al. identified three molecular subtypes via analyses of hybridisation array-based mRNA expression data from untreated, primary resected PC: a classical, quasi-mesenchymal (QM) and an exocrine-like subtype [44]. The classical subtype was characterised by the expression of the endodermal lineage-specifying transcription factor *GATA6, KRAS* dependency and better survival outcomes, whereas the QM subtype was associated with a high tumour grade and poor survival [44]. In 2015, Moffitt et al. identified two tumour subtypes (basal-like and classical) and two stromal subtypes (normal and activated) using non-negative matrix factorisation (NMF) and virtual microdissection of microarray and RNAseq data from primary and metastatic PC tumours [45]. The classical subtype was associated with a better survival outcome and overlapped with Collisson’s classical subtype. The basal subtype was characterised by the expression of known basal-like genes, such as laminins and keratins, worse survival outcomes and potentially a larger benefit from adjuvant chemotherapy. In 2016, Bailey et al. identified four stable classes using unsupervised clustering of RNA-seq data for 96 tumours with high epithelial content (≥40%), which were maintained in an extended set of mRNA hybridisation data for 232 PCs covering the full range of tumour cellularity (from 12–100%). Based on the expression profiles, the molecular subtypes were named squamous, pancreatic progenitor, immunogenic and aberrantly differentiated endocrine exocrine (ADEX) [33]. The squamous subtype was found to be enriched for gene programmes described in squamous-like tumours of breast, bladder, lung and head and neck cancers [46], including inflammation, the hypoxia response, metabolic reprogramming and TGF-β signalling, and was characterised by poor survival [33]. In contrast, tumours of the pancreatic progenitor subtype had a better outcome and were primarily defined by pathways involved in pancreatic endodermal differentiation. The ADEX subtype was described as a sub-class of pancreatic progenitor tumours, with transcriptional programmes characteristic of a more terminally differentiated normal pancreas. Lastly, by extending the analysis to the transcriptome of the immune infiltrate in the tumour microenvironment, the immunogenic subtype was identified with enrichment for pathways involved in immune cell infiltration and related immune signalling pathways [33].

Despite different technical approaches and different nomenclatures, there is substantial overlap between classifications, with the overall consensus that there are two major lineages, which are largely driven by epigenetic events that separate PC into a squamous/basal-like subtype and a classical subtype. The squamous/basal-like subtype is characterised by mutations in genes involved in chromatin modification, including DNA methylation and acetylation, such as *MLL2*, *MLL3* and *KDM6A* [33]. Tumours of this subtype have lost the expression of key genes involved in endodermal and pancreatic cell fate determination via DNA methylation, including GATA6 and HNF4A, which can distinguish them from the other molecular subtypes. Typical clinical aspects are a high tumour grade, metastatic disease, potential resistance to fluorouracil-based therapy, as shown in ESPAC-3 and COMPASS studies, and an overall poor prognosis [45,47,48,49,50]. The classical subtype is likely to contain a spectrum of tumours, including different immunogenic subtypes, and, overall, is characterised by a more favourable outcome. Moreover, classical and basal subtypes have been shown to co-exist within a tumour and be a consequence of a gene expression continuum, resulting from a mixture of expression programs from heterogenous cell populations, with the basal subtype becoming more dominant in metastatic disease [49,51].

Collectively, the molecular characterisation of PC and subtyping of the disease is providing a unique opportunity to identify new prognostic and predictive biomarkers to improve patient selection for treatments that target larger subgroups (Figure 1). Integrating transcriptomic data with genomic sequencing, protein assays, immune profiling or functional screens can identify additional clinically relevant subtypes [14,52,53]. For example, the squamous subtype has been shown to be enriched for replication stress, which may render these tumours sensitive to agents targeting the cell cycle checkpoint machinery [52]. However, how to successfully translate the multitude of molecular classifications described into the clinic using validated clinical assays remains an important area of research. A recent study assessing the robustness and clinical relevance of the three major subtype classification schemas described above [33,44,45] showed that a tumour-intrinsic two-subtype schema of classical and basal-like tumours is the most replicable. Moreover, the authors developed an assay (Purity Independent Subtyping of Tumours (PurIST)) that allows for subtype calling on multiple gene expression platforms, including microarray, RNA sequencing and NanoString, making it highly suitable for clinical use [54]. Alternatively, *GATA6* expression has been proposed as a surrogate biomarker to differentiate between classical and basal-like PC subtypes, which could reliably be detected using semiquantitative RNA in situ hybridisation or immunohistochemistry in the clinical setting [47,50]. Moreover, *GATA6*-low or basal-like tumours were found to be less responsive to FFX, and thus, have the potential to serve as a predictive biomarker of a therapeutic response, which is being prospectively evaluated in the Pancreatic Adenocarcinoma Signature Stratification for Treatment (PASS-01) trial (NCT04469556) [47,48,50,55].

## 5. Precision Medicine Initiatives

Compared to other cancers, precision medicine approaches that translate the increased knowledge of the molecular pathology of cancer into the clinic are in their relative infancy for PC. Several technical, organisational, and economic barriers hamper the realisation of personalised therapeutic development in patients with this tumour [56,57]. The anatomically difficult location to obtain sufficient material for analysis, together with the histopathological characteristics of PC (low cellularity, abundant stroma, intratumoural heterogeneity) represent major challenges to its biological characterisation [29,58,59,60,61]. In addition, the turnaround time of molecular analyses is often not compatible with the clinical behaviour of PC, which is notorious for the rapid clinical deterioration of the majority of patients. Lastly, organisational and financial issues need to be addressed, including the implementation of multidisciplinary teams specialised in PC care and research, investments in bioinformatic capacities and economic reimbursement for molecular testing [29]. Despite these challenges, recent years have shown a rapid expansion of multiple initiatives taking place globally, with encouraging experiences showing the feasibility and clinical benefit of genomic-based approaches [2,28,47,62,63].

Aguirre at al. showed the feasibility and merit of performing high-quality genomic profiling in clinically relevant timelines (<35 days) and identified potentially actionable somatic and germline alterations in 48% of 71 patients analysed, including DDR gene mutations, KRAS wild-type tumours, BRAF alterations and ROS1 translocation [2]. Moreover, (likely) pathogenic germline alterations were reported in 18% (13/71) of patients, which is a markedly higher percentage than would be expected based on family history. Genomic information was used in 30% of patients to drive personalised treatments, both in clinical trials and in off-label settings, with, for example, documented clinical response to MAPK pathway inhibition for two patients with oncogenic in-frame *BRAF* deletions [2]. Similarly, the COMPASS study (NCT02750657) demonstrated the feasibility of real-time WGS and RNA sequencing of advanced PC and its utility in identifying predictive mutational and transcriptional features for better treatment selection [47,50]. In addition to a differential response to first-line chemotherapy based on the molecular subtype or GATA6 expression (basal versus classical), potentially actionable genetic alterations were found in ≈30% of patients, including *BRAF* (2%), *CDK4/6* (7%), *PIK3CA* (7%), *PTEN* (5%) and *RNF43* (3%) mutations [47] (Figure 1). Data from the Know Your Tumor (KYT) initiative demonstrated the feasibility of screening PC patients in a community setting across the USA and allocating them to a matched targeted therapy [28]. Of 1856 patients with PC referred to the KYT programme, 1082 (58%) patients received a molecular profiling report, which included actionable molecular alterations in 282 (26%) cases. A retrospective analysis of patients for whom clinical follow-up was available showed that patients who received genomically matched therapy (*n* = 46) had a significantly longer median overall survival time compared to those who received unmatched therapies (*n* = 143; 2.58 versus 1.51 years) or compared to those who did not possess an actionable molecular alteration (2.58 years versus 1.32 years) [28].

Altogether, these studies demonstrated that a genomics-driven precision medicine strategy can be safely integrated into current clinical management with a rapid turnaround time and has a high potential to drive both current and novel investigational therapeutic choices. However, data remains largely anecdotal or retrospective, with only a small number of patients receiving molecularly matched treatment, and major efforts are needed to conduct biomarker-directed clinical trials that are adequately powered for small groups of patients carrying a diverse range of potentially actionable genetic aberrations.

## 6. New Models of Therapeutic Development

**Figure 2:**
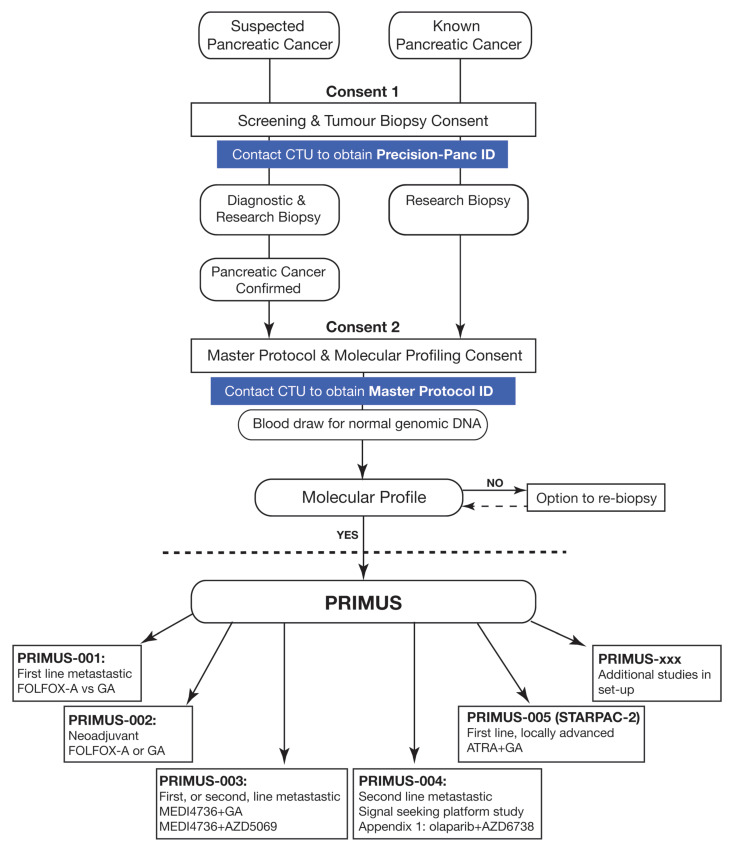
Precision-Panc Master Protocol and different Pancreatic canceR Individualised Multi-arm Umbrella Study (PRIMUS) studies. CTU (Clinical Trials Unit), FOLFOX-A (FOLFOX and nab-paclitaxel), GA (gemcitabine and nab-paclitaxel), ATRA (all-trans retinoic acid).

To overcome the challenges that are inherent to precision medicine and the low frequency of molecular alterations of interest, novel approaches using adaptive statistical designs and a master protocol to assign patients to different candidate drugs has shown promise in many tumour types [64]. The Investigation of Serial Studies to Predict Your Therapeutic Response with Imaging and Molecular Analysis (I-SPY) series for breast cancer [65,66], the National Lung Matrix Trial for non-small cell lung cancer [67] or basket trials that select tumours according to their molecular characteristics irrespective of tumour type, such as the NCI-MATCH (NCT02465060) initiative [68], are only a few of the many examples. Overall, this methodology allows for the evaluation of multiple hypotheses of sub-studies conducted in selected subgroups, increases patient study eligibility and thereby offers more attractive clinical trial options for patients. In this context, multiple therapeutic development platforms are starting to develop for PC, including Precision Promise (NCT04229004) or the PASS-01 (NCT04469556) trial in the USA and Canada, or Precision-Panc in the U.K. [69]. By integrating the discovery, preclinical and clinical development pillars of therapeutic development, Precision-Panc aims to improve patient outcomes through the rapid translation of preclinical advances into a diverse range of PRIMUS (Pancreatic canceR Individualised Multi-arm Umbrella Study) trials. To tackle the issues specific to PC, as highlighted above, a master protocol serves as a multicentre “portal” protocol for flexible tissue acquisition and molecular profiling of patients with known or suspected PC (Figure 2). Patients who undergo endoscopic ultrasound (EUS) or a radiologically guided biopsy for suspected PC are informed about the study and provide consent for enrolment in the Precision-Panc Master Protocol (stage 1 consent). At that stage, extra tissue and a peripheral venous blood sample are taken for molecular profiling at the same time as the diagnostic workup. Once a diagnosis of PC is confirmed, a stage 2 consent is required to proceed with molecular profiling, including DNA and RNA extraction from the surplus diagnostic tissue and germline DNA analysis from a venous blood sample. Targeted capture sequencing is performed using the Glasgow Precision Oncology Laboratory (GPOL) Clinical Cancer Genome (CCG)^TM^, which is a well-designed, pancreatic cancer-specific multiplex assay that includes point mutations, copy number, structural variations, fusions and tumour mutational signatures. This approach has been shown to deliver successful molecular profiling results for all stages of PC in a clinically relevant time frame, including samples obtained using EUS, with a low failure rate of only ≈2%, without the need to for additional visits or procedures [70]. The results of molecular profiling obtained through the Precision-Panc Master Protocol may subsequently provide information regarding the eligibility for enrolment in a PRIMUS study by examining different biomarker-based treatment regimens. By combining molecular profiling with clinical response data and rapid forward and backward translation between the laboratory and the clinic, multiple hypotheses will be tested that allow for the discovery of therapeutic responses, candidate biomarkers and mechanisms of resistance. From its inception in December 2017, Precision-Panc clinical trials are available in 25 NHS sites with another 9 being set up. There are more than 300 participants enrolled in the 3 initial trials on offer, with additional studies anticipated to open in the near future to work towards the overall aim to have a trial option for each patient (Figure 2).

## 7. Conclusions

The extensive molecular variability within PC revealed by recent multiomics studies requires a paradigm shift from the standard histopathological classifications to a new meaningful and clinically relevant molecular taxonomy available for all patients at the moment of initial diagnosis. Fundamental in our efforts to improve outcomes for PC patients is the integration of pre-clinical and clinical research with continuous forward and backward analyses to identify the key molecular processes that determine certain vulnerabilities and therapeutic responses. Here, we have merely focussed on precision medicine approaches based on genomic and transcriptomic subgroups. However, targeting the tumour stroma, as well as providing an integration of multiomics and functional studies into, for example, the altered epigenome, the immune landscape or distinct patterns of metabolism, is likely to reveal additional important therapeutic vulnerabilities, as reviewed elsewhere [71,72,73]. A dynamic therapeutic development platform, such as Precision-Panc or similar initiatives elsewhere, allows for addressing all these elements and represents a unique opportunity to define a new molecular taxonomy that serves to guide and realise the promise of precision medicine for PC patients.
